# Book Reviews

**Published:** 1861-07

**Authors:** 


### OUR LIBRARY TABLE.

*Die Brandstiftungen in Affecten und Geistesstörungen. Ein Beitrag zur gerichtlichen Medicin für Juristen und Aerzte.* Von

Dr. Willers Jessen, 8vo. pp. 335. Kiel, 1860. *Incendiarism as depending upon the Passions and the various forms of Insanity.*—This is a large book upon a subject which might seem capable of being disposed of in an article of a journal, as indeed it was the author's original intention it should have been. He is well entitled to the credit he lays claim to for the laborious industry manifested in its compilation. Every one is aware, he observes, of the discrepancy of opinion which prevails upon the subject of incendiarism-mania, some maintaining and others stoutly denying its existence, and both parties basing their opinions upon facts, cases, and decisions. The opponents of its reality not only deny the sufficiency of the facts relied upon in proof of this, but themselves adduce cases of incendiarism in which no such mania existed—a negative procedure of very little utility, inasmuch as the fact of its non-existence in some cases is no proof that it may not prevail in others. The best means of deciding the dispute would seem to be the collection and critical examination of as many cases of incendiarism as could be obtained, and this is the task which the author set himself. Abundant material was thus obtained; but from amidst this, those cases had to be selected which were described with sufficient precision to allow of the detection of the motive and psychical process which had led to the action, and consequently the psychical condition of each malefactor. Of the cases so collected those which were due to passions or emotions, as revenge, fear, &c., were first separated from the cases in which some form of diseased mind could be traced, a certain number of the cases, however, seeming to form a connecting link between the two divisions. During this preliminary inquiry the question of the specific nature of pyromania had to be laid aside, and it soon appeared that this was a matter of no great signification. It was found that strong and determinate, which may therefore be termed specific impulses to pyromania resulted from various psychical processes, and that a number of different diseased conditions occurred which might be termed pyromania, but that there was no proof of a specific incendiary monomania or pyromania. The fundamental question is, what are the various general psychical conditions and processes that give rise to this impulse to incendiarism; and the object of this book is to exhibit these different circumstances. This is done, as we have already said, in great detail, each case being minutely analysed in order to trace out the nature of the motive or of the diseased condition upon which the action was dependent. In this way the author has assembled an instructive repertory of more than a hundred cases, which, although they have all been published before, have now the advantage of being collected together from scattered sources, and of being submitted to a critical examination by a competent hand.

*On the Surgical Diseases of Women.* By I. Baker Brown, F.R.C.S. Second edition, revised and enlarged. 8vo, pp. 410. London, 1861 (Davies).

The profession will heartily welcome a second edition of this valuable work. In its present form it contains much additional matter, more particularly upon the following subjects:—Intra-Uterine Fibrous Tumours, Hypertrophy and Irritation of the Clitoris, Cauliflower Excrescence of the Uterus, Certain Diseases of the Rectum producing or simulating Uterine Disorder, Certain Surgical Lesions connected with Sterility, Ruptured Perinæum, Vesico-Vaginal and Recto-Vaginal Fistula, and Ovarian Dropsy.

One of the most notable and valuable features of the work is the copiousness of the clinical illustrations, so that the author is justified in stating in his prefatory observations, that he puts "the chief merit of the book in its being a mirror of clinical observation—a practical treatise on some most important surgical operations, the value of which is discoverable in the record of appended cases." Further he says, "I would commend this treatise to the members of the medical profession, not as a recondite treatise on the pathology of the several affections considered in its pages, but as a contribution to practical surgery; and with the hope that its publication may serve to advance and perfect the means of cure for a number of diseases ranking among the most painful to which woman is subject."

The author has fully justified this commendation, a fact which best indicates the high value of his work.

*Elements of Medical Zoology.* By A. Moquin-Tandon, Professor of Medical Natural History to the Faculty of Medicine at Paris. Translated and edited by Robert Thomas Hulme, M.R.C.S.E., F.L.S. London, 1861. (Baillière.) Small 8vo, pp. 41.

On the whole, we must own to a prejudice against mongrel works of this class, but on looking over M. Moquin-Tandon's Manual, we frankly confess that it is one calculated to be of great utility to many students, medical and pharmaceutical, and practitioners, and that Mr. Hulme has rendered a service to our medical literature by translating it—a service heightened by the judicious manner in which, in the exercise of his editorial functions, he has enriched the text with additional matter.

The author has, in this work, adopted an arrangement founded upon the characters of the animal or its Medico-Zoological relations. "Such an arrangement," as he observes, "is more practical than scientific; but it is simple, convenient, and well adapted for the purposes of medical or of pharmaceutical study, and avoids leading the reader into details which are foreign to his daily occupation." He adds, "Many animals and many animal productions, which were formerly in use, are no longer employed in medicine. These might have been omitted, but as it is useful to have a knowledge of the Ancient Materia Medica, and to be acquainted with the history, the revolutions, and the progress of therapeutics, I have given a short description of these animals and of their productions in a separate chapter." Medical Zoology, of necessity, also includes the Natural History of Man.

The manner in which both author and translator have effected their work will be best seen by one or two illustrations.

In the chapter on the Unity of the Human Species we read—

"Some naturalists have endeavoured to establish several distinct species of men.

"Linnæus in his *Systema Naturæ* (1766) admits two species of men, the *Homo Sapiens* and the *Homo Troglodytes*. Under the latter title he includes the *Albino*; these, however, are persons in a state of disease, and in the present day they are not regarded as even constituting a variety. Linnæus imagined that this supposed second species lived in caverns, and for this reason he bestowed upon it the name of troglodytes, characterising it by the epithet of nocturnal (*nocturnus*). At the end of his *Mantissa plantarum altera*, which appeared five years after the twelfth edition of the *Systema Naturæ*, the illustrious naturalist of Sweden committed the serious error of including in the genus *Homo* an ape, the *Gibbon* of Buffon, which he names *Homo Lar*; 'a surprising error committed by a great genius, which should never find imitators.' (Pouchet.)

"Virey (1821) also admitted two species of men, distinguished by the difference of aperture in the facial angle; in the one it varies between 85° and 90°; in the other between 75° and 82°. In the apes it never exceeds 40°. These two species of men include six races characterized by their colour, and these again comprise eleven sub-races, which are arranged according to the regions they inhabit.

"Desmoulins (1824) divided the genus man into eleven species more or less distinct; the characters he gives them are often established with considerable ability, but they are always insufficient to induce us to reject the unity of the human race. He names these species:—1st, the *Celto-Scyth-Arabs*; 2nd, the *Mongols*; 3rd, the *Ethiopians*; 4th, the *Euro-Africans*; 5th, the *Austro-Africans*; 6th, the *Malays* or *Oceanians*; 7th, the *Papous*; 8th, the *Negro-Oceanians*; 9th, the *Australasians*; 10th, the *Columbians*; 11th, the *Americans*.

"Bory de Saint-Vincent (1825) goes even farther than Desmoulins: he admits fifteen species of men. These are:—1st, the *Japetic*; 2nd, the *Arabian*; 3rd, the *Hindoo*; 4th, the *Scythian*; 5th, the *Sinic* (Chinese); 6th, the *Hyperborean*; 7th, the *Neptunian*; 8th, the *Australasian*; 9th, the *Columbian*; 10th, the *American*; 11th, the *Patagonian*; 12th, the *Ethiopian*; 13th, the *Caffre*; 14th, the *Malanian*; 15th, the *Hottentot*. He arranges these fifteen species into two tribes: 1st, the LEIOTRIX, or those with smooth hair; this division includes the *Japetic*, *Arabian*, *Hindoo*, *Scythic*, and *Sinic* species belonging to the Old World; the *Hyperborean*, *Neptunian*, and *Australasian* species, common to the Old and the New, and the *Columbian*, the *American*, and the *Patagonian* species, peculiar to the New World; 2nd, the OULOTRIX, or those with crisp hair, containing the *Ethiopian*, *Caffre*, *Malanian*, and *Hottentot* species.

"In the present day man is generally regarded as constituting a simple species, in which all the individuals are capable of mingling indiscriminately, and are able to produce an offspring which is as fruitful as its parents."

Again, from the section devoted to Animals or Animal Productions formerly employed in Medicine, we learn that—

"The ancient therapeutists often sought for what they termed correspondence between the disease and the remedy, but it is impossible to conjecture what were the relations upon which they founded the virtues of many animal substances. Thus in spitting of blood, they recommended the patient to drink kid's blood mixed with vinegar; in diseases of the kidneys they prescribed the back of a hare to be eaten raw or cooked, *but without touching it with the teeth*; in diseases of the spleen, they applied the spleen of a dog over the region of the affected organ; in disorder of the liver, they ordered the dried liver of a wolf in wine sweetened with honey, or that of an ass bruised in honey with two parts of celery and three nuts!

"The following are some of these therapeutic agents which belonged to the ancient medical zoology, arranged in three series :—

"I. THE ENTIRE ANIMAL.

1st. *Simply opened or bruised.*—Bat, mole, pigeon, toad, tree-frog, spider, scorpion.

2nd. *Dried or reduced to powder.*—Hedgehog, titmouse, water-wagtail, wren, goat sucker, plover, snake, toad, earthworm, bug, cricket, grasshopper, ant. . . . .

3rd. *Calcined and reduced to ashes.*—Badger, mouse (*mus combustus*), crow, cuckoo, kingfisher, lizard, salamander, slug, scarabæus.

4th. *Infused in water.*—Magpie (*aqua picarum compositum*), swallow (*aqua hirundinum*).

5th. *Boiled in milk.*—Toad.

6th. *Infused in oil.*—Dog (*oil of young dogs*), fox, hawk, cameleon, scorpion (*oil of Matthiole*), cockroach, earthworm.

7th. *Distilled.*—Ants (*water of magnanimity*).

"II. BONES of the dog, wolf, hare (*astragalus*), horse, stag, eagle (*skull vertebræ*), toad (*left humerus*), carp, shad, and whiting.

"III. BLOOD of the bat, lion, dog, mole, weasel, hare, rat, horse, ass, elephant, rhinoceros, bull, camel, stag, goat, goldfinch, lark, pigeon, cock, pheasant, quail, ostrich, swan, duck, tortoise, lizard, frog, tree-frog, and snake.

"IV. FAT of monkey, dog, wolf, fox, wild cat, hedgehog, badger, rabbit, hare, marmot, beaver, porcupine, dormouse, ass, elephant, stag, fallow-deer, camel, eagle, falcon, kite, common fowl, pheasant, cassowary, heron, frigate bird, pelican, lizard, snake, frog, tree-frog, carp, pike, eel-pout, and lamprey.

"V. COVERING.

1st. *Skin* of mole, horse, ass, rhinoceros, eagle, tench, and eel.

2nd. *Hair* of cat, fox, hare, horse, ass, elephant, goat, camel. .

3rd. *Feathers* of eagle, lark, partridge.

"VI. SHELLS.

1st. *Univalves*—snail, rudimentary shell of slug, whelk, dentalium.

2nd. *Bivalves*—common mussel.

3rd. *Epiphragma* of the large Roman snail.

4th. *Pearls* of the pearl oyster and the mussel.

"VII. NUTRITIVE ORGANS.

1st. *Jaws* of the pike, trout. .

2nd. *Teeth* of wolf, badger, wild boar, cod, &c.

3rd. *Tongue* of grouse, flamingo. .

4th. *Stomach* of hedgehog, pigeon, common fowl, crane, ostrich, eel-pout. .

5th. *Intestines* of wolf. .

6th. *Spleen* of dog, ass. .

7th. *Liver* of wolf, mole, bear, badger, weasel, otter, hare, porcupine, elephant, goat, roebuck, eagle, swan, duck, lizard, frog, eel. .

8th. *Kidneys* of ass. .

9th. *Lungs* of fox (*pulmones preparati*), weasel, hare, pig. .

10th. *Heart* of monkey, lion, mole, stag, crow, peewit, kingfisher, toad." .

In an equally interesting and serviceable manner the excrementitious matters, and the different parts of the organs of relation and reproduction, &c., are summed up.

The following account of the *Tsetse* will afford an excellent illustration of the more strictly zoological portions of the work :—

"The *Tsetse* or *Tzetse* is a very formidable Fly which inhabits Africa. Bruce, who met with it in Abyssinia, has given a bad drawing of it, but has correctly described its habits.

"MM. Arnaud, Livingstone, Oswald, L. de Castelnau, and Anderson, have collected many curious details concerning this insect. Mr. Westwood has given a very good description of it.

"The *Tsetse* belongs to the genus *Glossina.* It is named the *Biting Glossina, Glossina morsitans,* Westw.

"Nearly all the central countries of South Africa are more or less infested by the *Tsetse;* it is very common in all the countries situated to the north of Lake Ngami; and is again met with in Soudan and in the tropical districts.

"This insect usually frequents the bushes and reeds on the borders of marshes. It is larger than the common fly, and of a whitish yellow colour; the thorax is of a pale chesnut on its upper surface, is covered with grey hairs, and has four longitudinal interrupted black bands in the centre; its proboscis is twice as long as the head, and is extremely slender; it resembles a fine corneous thread; the palpi are straight, of the same length as the proboscis, and form a sheath for it; the abdomen is a light yellow with darker spots or bands; the wings are smoke-coloured.

"The buzzing of the *Tsetse* is a mixture of a dull and a sharp sound, producing a very discordant noise; this buzzing spreads a terror and disorder amongst men and animals which even the wild beasts of the country when they are twice their number will not produce. (Bruce.)

"Its vision is extremely acute, and it darts like an arrow upon the animal that it intends to attack; it always makes its puncture between the belly and the thighs, when a swelling soon rises up around the wound.

"The horse, the ox, and the dog, after they have been attacked by this insect, waste away and die in the course of a few days; those which are fat and in good condition, soon die, while the others drag on a miserable existence for some weeks; three or four flies are sufficient to produce these disastrous results. The blood of the animals which die is altered and diminished in quantity; the fat in the neighbourhood of the wound is soft, viscous, and of a yellow colour; in general, some portion of the intestines is enormously swollen; the flesh putrifies very quickly (Castelnau); and the heart, the lungs, and the liver are more or less affected. The goat is the only domesticated animal which can live with impunity in the midst of these flies; dogs escape the danger when they are fed exclusively by means of the chase, but if these animals are fed with milk they invariably die; on the contrary, the calf has nothing to fear so long as it sucks.

"The bite of the *Tsetse* is not dangerous to the wild animals; the elephant, zebra, buffalo, and the various kinds of antelopes and gazelles which abound in the countries inhabited by this fly, do not experience any ill effects from it.

"These insects do not bite when it is bright moonlight, or when the nights are very cold.

"*Action on man.*—The *Tsetse* also attacks our species, but its action on man is attended with but little danger; its bite is very analogous to that of the gnat's, but the pain does not last so long. (De Castelnau.) M. Arnaud, however, suffered for some months after being bitten by one of these insects.

"M. Chapman, one of those who have penetrated the furthest into the interior of South Africa, states, that whilst he was hunting, having a small hole in his dress made by a pin, he has often seen one of the *Tsetse,* which appeared to know that it could not penetrate his dress, dart down, and, without ever missing its mark, wound him through the undefended opening.

"Is the *Tsetse* a poisonous animal? Its effect on the domesticated animals would appear to answer the question in the affirmative, but its action on man declares the contrary. How then are we to explain its fatal effects on cattle?

At the same time these results vary in different species, and in some they are of no consequence."

*Epileptic and other Convulsive Affections of the Nervous System, their Pathology and Treatment.* By CHARLES BLAND RADCLIFFE, M.D., F.R.C.P., Physician to, and Lecturer on Materia-Medica and Therapeutics at, the Westminster Hospital, &c. Third Edition. London: Churchill. 1860. Small 8vo. pp. 312.

In the third edition of this well-known work Dr. Radcliffe has incorporated the Gulstonian Lectures, which he delivered at the Royal College of Physicians, in the spring of 1860. He has also re-written and re-cast his materials "so as to make a new book rather than a new edition." Dr. Radcliffe, as will be familiar to our readers, seeks to establish an entirely new theory of muscular action, which, being confirmed, would revolutionize our notions upon the pathology and treatment of convulsive disorders. In the previous series of this Journal, on the publication of the second edition of Dr. Radcliffe's work, we entered largely into the consideration of his physiological theory,* and the favourable opinion we then formed of it, as well as the high value we attached to its bearing upon pathology and treatment, are abundantly confirmed by the maturer arguments contained in the present volume. He holds that, "In opposition to the theory which supposes that muscle is endowed with a vital property of contractility, and that contraction is brought about when this property is roused, or excited, or *stimulated* into action, it may be held that there is a state of polarity in living muscle during relaxation which produces relaxation, and that contraction is nothing more than the necessary result of the muscle being liberated from this state and left to the operation of the attractive force which is inherent in the physical constitution of the muscular molecules."

In illustration of the singularly suggestive character of the present volume, we may quote the following observations on the part the medulla oblongata plays in epilepsy :—

"It is evident, then," writes Dr. Radcliffe, commenting upon Dr. Van der Kolk's researches, "that the medulla oblongata is especially affected in epilepsy; but it does not follow, as Dr. Van der Kolk supposes, that the essential cause of the convulsive affection is to be found in an exalted sensibility and activity of the ganglionic cells of this centre.

"In favour of this view,—that epilepsy is dependent upon exalted sensibility and activity of the ganglionic cells,—this physician appeals to the fact of spasm, to the presence of a full, bounding pulse, and to the freedom from attack which is for some time the fruit of an attack, particularly if this has been violent; but it is easy to see that this appeal is one which cannot be allowed. After what has been said about muscular motion, it is not possible to allow that spasm is an argument in favour of exalted sensibility and activity in ganglionic cells. After what has just been said about the phenomena of the circulation in epilepsy, it is not possible to allow that the full and bounding pulse of the epileptic paroxysm is produced by increased injection of arterial blood into the vessel. If this could be allowed, there would be no difficulty in supposing that this

* See *Journal of Psychological Medicine*, vol. xii. p. 89.

pulse must imply exalted action of the medulla oblongata—for the action of any organ must be in direct relation to the amount of arterial blood supplied to it; but, so far from this being the case, the simple fact is that the full and bounding pulse of the epileptic paroxysm is the pulse of suffocation—a pulse which is filled with black blood, not with red. And thus the notion of increased injection of arterial blood during the epileptic paroxysm falls to the ground, and with it the theory of exalted activity of the medulla oblongata, which has been based upon it. Nor can the freedom from attack, which is for some time the fruit of an attack, be appealed to as a certain proof that the attack is the sign of the discharge of some overcharge of excitability previously present. On the contrary, it may be argued with some degree of plausibility, from certain facts which have to be mentioned hereafter, that the attack was preceded by depression of the circulation and innervation, that the convulsion supervened when this depression had reached a certain point, and that the recurrence of the attack was prevented for a time by the state of reaction in the circulation and innervation, which is a consequence of the convulsion. The case may be one, indeed, of which the history of the rigors of ague may serve as no inapt illustration; for here we have, first, the circulation failing more and more until the bathos of the cold stage is reached, and then a state of reaction which banishes the rigors most effectually so long as it continues.

"It would even seem as if appeal might be made to the appearances after death, and to the actual condition of the circulation in the fit, for positive arguments against the idea of anything approaching to exalted action of the medulla oblongata.

"The signs of fatty degeneration can have but one significance—under-action, not over-action. The interstitial deposit, also, implies an equivalent absence of healthy nerve structure, and so does the dilated condition of the blood-vessels; and this absence of nerve structure must necessitate a corresponding absence of nervous action. The appearances after death, indeed, if they show anything, would seem to show that the medulla oblongata of the epileptic is *damaged in structure*, and because damaged in structure, *weaker in action* than it ought to be.

"The great argument against the idea of anything like over-action of the medulla oblongata in epilepsy, however, is to be found in the state of the circulation; for if, as may safely be assumed, the activity of any organ is in direct relation to the activity of the circulation of *red blood* in that organ, how far from anything like over-action must be the state of things in which, as is the case in the epileptic paroxysm, the vessels are at first comparatively empty of red blood, and afterwards completely filled with black blood?"

Dr. Radcliffe next shows that Dr. Brown-Séquard's experiments on the spinal cord cannot be "construed into an argument that there is anything like a state of exalted action of the *spinal cord* in epilepsy," and he then proceeds:—

"But, it may be asked, is there nothing else? Is there no peculiar state of the nervous system in epilepsy? Is there no '*morbid irritability?*' In order to answer this question, it is necessary to ask another—What is 'morbid irritability?' It is not inflammation; it is not fever; it is some indefinable and negative state which occurs frequently in teething, in worm disease, in uterine derangement, and in many other cases—a state in which the patient is unusually depressed by depressing influences, and unusually excited by exciting influences. But what is this state? Is it anything more than mere exhaustion? In difficult teething, the strength is worn away by pain and want of sleep; in worm disease, the parasites help to starve and exhaust a system already ill fed and feeble; in uterine derangement, the health is undermined, in all probability, by pain and by sanguineous or other discharges. In each case there is unequivocal exhaustion of body and mind, and the signs of 'morbid irritability' appear to be nothing more than the signs of such exhaustion. A weak person is more affected by the several agencies which act upon the body from within and from without, and he is so because he is without some of that innate strength which belongs to the strong person; and the person who is 'morbidly irritable' is, in reality, one who, for want of this principle of strength, is inordinately excited or depressed by the several exciting or depressing influences which are continually acting upon the system. In a word, this undue 'morbid irritability' may be nothing else than the natural consequence of that general want of power, the signs of which are written so legibly upon the vascular and nervous systems of the epileptic. Thus interpreted, indeed, 'morbid irritability' only becomes another name for inefficient innervation.

"In these points of view, therefore, *the pathology of ordinary epilepsy* would seem to be altogether unintelligible upon the current theory of muscular motion—a theory according to which the muscles are supposed to contract convulsively, because they are subjected to excessive stimulation. In these points of view, indeed, the pathology of this disorder would seem to be only intelligible when it is interpreted by the theory of muscular motion which is advanced at the commencement of this volume."

In the medicinal treatment of epilepsy, Dr. Radcliffe makes the following highly important suggestions, which alone suffice to stamp the great practical value of his work.

"Reflecting upon the physiological and pathological premises, the great desideratum in a case of epilepsy would seem to be a more vigorous action of the nervous centres, for, according to these premises, convulsion itself is the consequence of a failure in this action; and hence it may be supposed that the remedy which has yet to be applied is one which will have some special power of rousing and sustaining the action which is deficient. Reflecting upon what has already been said respecting the beneficial influence of cod-liver oil in epilepsy, and upon the possibility that this remedy may do good by furnishing one of the materials, namely, the fat, which is necessary to the full nutrition of the fatty nervous tissue, it may be supposed that the remedy which has yet to be applied is one which will supply this kind of food to the nervous system. But is this all? Is there any material besides fat or oil which may be wanted to secure the healthy nutrition of the nervous system? These questions suggest themselves naturally; for however mysterious the properties of the nervous system, this much is plain enough—that the nervous tissue cannot exist independently of proper nutrition, and that any means which will merely rouse its action, without at the same time providing for its nutrition, must in the end do harm, and not good. Now, on examining the chemical constitution of nervous tissue, it is evident that phosphorus is a very important ingredient. It is evident, too, that the amount of fat and phosphorus has some relation to the activity of the nervous centres, for both these ingredients increase from infancy to adult age, and decrease afterwards as the influence of advancing age tells upon the system. It is also a most significant fact that the proportions of fat and phosphorus approximate very closely in infants and idiots. . . . . The ingredients of the spinal cord and of the nerves are substantially the same as those of the brain, and there is reason to believe that the proportions of fat and phosphorus vary in the same manner at different periods of life. Here, then, are some very curious and striking facts. Here is nearly two per cent. of phosphorus in the brain of adults, and in infants and idiots considerably under one per cent. Here is, that is to say, little more than half the proper proportion in a state between which and the worst forms of epilepsy there is a somewhat close relationship. The facts, indeed, are well calculated to suggest the question whether phosphorus may not be as necessary as fat to the proper nutrition of a weak nervous system—as necessary as iron where there is a deficiency of red corpuscles in the blood; and this question, once put, would seem to require an answer in the affirmative. 'In *small doses*,' says Dr. Pereira, 'phosphorus excites the nervous, vascular, and secretory organs. It creates an agreeable feeling of warmth in the epigastrium, increases the fulness and frequency of the pulse, augments the heat of the skin, heightens the mental activity and the muscular powers, and operates as a powerful sudorific and diuretic.' In large doses, phosphorus, without doubt, is a caustic poison; in proper doses it produces the very changes which are necessary in a case of epilepsy. In proper doses, and under the eye of a medical man, it is quite innocent of harm, and it may be productive of much good.

"This inference is that which may be drawn from what has been said; and this inference, so far as I can see, is not contradicted by experience. Given in the large doses in which phosphorus has been given in a few cases already on record, the good resulting may have been doubtful—very doubtful; but this experience is nothing to the point, for there is no reasoning in any case as to the effects of medicinal doses from the effects of poisonous doses. Given in medicinal doses, I have seen enough to know that this remedy may be given, not only without harm, but with the unmistakeable promise of real and substantial good. As yet my experience is very limited, for it is only the other day that the idea of using oil and phosphorus as nervine tonics occurred to me; but, as I have just said, I have already seen enough to justify me in saying what I have just said. I have also seen what leads me to expect that the promise of good from this mode of treatment is not confined to cases of ordinary epilepsy, but that it includes other cases in which the action of the nervous centres, one or more, is defective—namely, two cases of hysterical paraplegia, one case of weakness of the arm and leg on one side following some choreic symptoms, and one case of melancholia. The form in which I have given the phosphorus is the phosphorated oil of the Prussian Pharmacopœia—a preparation which is made by dissolving twelve grains of phosphorus in one ounce of almond oil by the aid of warm water. About four grains of the phosphorus are taken up, and the usual dose is from five to ten minims. I have given this phosphorated oil along with cod-liver oil, in a little orange wine, twice or thrice a day, the formula being—

"℞ Olei Phosphorati (Ph. Borussicæ), ♏ v—x;

Olei Morrhuæ, ʒii—iv. M."

*On Epilepsy and Epileptiform Seizures: their Causes, Pathology, and Treatment.* By EDWARD HENRY SIEVEKING, M.D., F.R.C.P., Physician to, and Lecturer upon Materia Medica at, St. Mary's Hospital. Second Edition, revised and enlarged. London: Churchill. 1861. Small 8vo. pp. 336.

Dr. Sieveking's and Dr. Radcliffe's works should be placed upon the same shelf. The former is the best systematic treatise we possess on the subject in the English language, the latter the most ingenious attempt to penetrate the mystery and construct a theory of the subject, and the one most fertile in practical results.

The present enlarged edition of Dr. Sieveking's work will add to the reputation of its author, and confirm the work in the favour of the profession—if, indeed, such confirmation were necessary.

The following illustration of the conjunction of hallucinations with epilepsy, given by Dr. Sieveking, will be read with painful interest:—

"Since the publication of the first edition of this work, the profession has lost one of its brightest ornaments, Professor Alison, of Edinburgh, in whose case hallucinations and delirium were associated with epilepsy. In the account given by Dr. Patrick Newbigging of the illness and death of this eminent physician and teacher, we find that his first seizure in 1846 was followed by delirium of a violent, and, for such a man, highly demonstrative character. The fits recurred at intervals of six weeks till 1850; they then became more frequent and severe, some being followed by headache and nervous excitement. In 1854, after a severe seizure in the month of October, Dr. Alison suddenly became delirious; the delirium passed off on the following day, and the patient recovered his usual state of health. During the next five years the frequency of the fits fluctuated a good deal. 'The nature of the terrible attacks,' to employ the author's words, 'to which Dr. Alison had become so great a martyr, gradually underwent certain changes. They became less numerous, but the fits were greatly increased in severity, and during this year the condition of my patient after the fits was very formidable and most painful to witness—the delirium assuming more and more the maniacal character, and latterly the difficulty of restraint, even with the assistance of two or three male attendants, was very considerable.' Towards the close there were frequent changes from comparative quietness to complete maniacal excitement until within a few days of dissolution, when Dr. Alison's state was thought to resemble that of a person sinking under typhoid fever. 'There were brief intervals towards the close,' says Dr. Newbigging, 'when our patient was sensible, was able to express his thanks to those around him in his usual calm manner, and when he stated his belief that his end was approaching. But these peaceful moments were soon followed by excitement characterized by violent spasmodic action and screaming, accompanied by a peculiar rotatory movement of the hands, or by a condition very frequent in the earlier attacks, at a certain stage of the seizure, when he seemed lost in the contemplation of some blessed vision, during which he expressed his belief that he heard the praises of the heavenly hosts, and that among the number he distinctly recognised the voices of dear departed friends.'"

After detailing the opinions of Brown-Séquard, Kussmaul and Tenner, and others upon the nature of epilepsy, Dr. Sieveking thus sums up his matured opinions:—

"I must leave the opinions and statements of the distinguished physiologists to whom I have referred in the preceding pages, to the judgment of the scientific world at large. It is not for me to do more than thank them for the light they have, each in his way, shed upon the labyrinth in which so many have lost themselves. But I cannot but dwell upon the fact, that they all agree with regard to the essential identity of the various forms of epilepsy, which have been distinguished from one another according to the nature of the exciting cause. Whether the tubercula quadrigemina, the pons and crura cerebri, or the medulla oblongata, be regarded as the main seat of the disease, the irritation conveyed to them,—through the mind, from any other part of the nervous centres, from another viscus, or from a cutaneous nerve,—equally excites the paroxysms through the agency of the same part of the cerebrum. The real difference between different forms of epilepsy consists in the different excitability of the individuals; in one it is such that the perverted action once having been set up, it exhibits a self-multiplying power which cannot be controlled; in another the excitability is so slight that the removal of the particular stimulus puts an end to all display of that excitability; while again, in a third, the excitability is moderate, but persistent, and liable to be called into action by any tolerably strong stimulus that may at any time be offered. It has been said, and I think with truth, that the possibility of producing epilepsy in every individual only depends upon the possibility of discovering the particular kind of stimulus which shall in him rouse his excitability to a certain point.

"Given the peculiar sensitiveness of the cephalic centre, which, on the application of a certain irritant, induces the paroxysm, what are the channels through which the irritant operates? Certainly in most, if not in all cases, it acts through the blood. A mere change in the balance of the circulation suffices in many instances; and if to that change a change in chemical constitution be added, the complication renders the physician's duty the more difficult. I might quote many cases illustrative of both these aspects. The following brief sketch of one may serve to show how epilepsy may depend upon an alteration in the balance of the circulation.

"A young lady consulted me, through the mediation of Mr. Spencer Wells, in whom I could not but regard the disturbance in the balance of the circulation as the main element in the production of the epilepsy to which she had been subject for three years and a half. She is now (June, 1857) sixteen years of age, and has always enjoyed admirable health, with the exception of the fits, which possess the pathognomonic features of epilepsy. After a minute and searching inquiry, the only fact of any importance discoverable in connexion with the disease is that, although an older sister had menstruated at thirteen, she herself has not as yet manifested any symptoms of the catamenial function; and at the time at which she would have menstruated had she followed her sister's example, but at which in her the fits made their appearance, the occasional attacks of epistaxis ceased to which she had been previously liable. One could scarcely avoid, in this instance, arriving at the conclusion that an *error loci* afforded the therapeutic indication; and that the disturbance in the circulation, acting upon a susceptible nervous system, deranged the polarity of the latter. As yet no material derangement of the nervous and intellectual functions are manifested; and were, from other causes, death to occur suddenly at this time between the paroxysms, I doubt whether it would be possible to detect any kind of lesion indicating an altered nutrition of the cephalic centre.

"I believe that, in the great majority of instances of epilepsy, the first attack is due to an irritation produced by derangement in the amount or quality of the blood circulating in the brain. In a person predisposed we frequently find over-fatigue, a long walk, carrying heavy loads, prolonged mental exertion, the manifest cause not only of the first, but of many succeeding seizures. Hence there will be occasion, in discussing the treatment of the disease, to dwell much upon the necessity of bodily and mental rest, so as to allow the system to recover that balance, the disturbance of which gave rise to the seizure. This is more marked in some cases than others; but in none can our remedial agents be attended with any beneficial result unless we have a regard to this important indication."

*Diagnostics of Aural Disease.* By S. E. Smith, Esq., M.R.C.S Eng. London: Baillière. 1861. 8vo. pp. 106.

This is a skilfully written work, adapted as well for the removal of popular errors on the subject among the public as for the profession. The reasons for its publication are best expressed by the author himself in the following telling observations:—

"We confess ourselves at some loss to what cause to impute the fact that so little has been done to diffuse a general knowledge of the diseases to which humanity is prone. In an age when so much is being accomplished forthe diffusion of knowledge on almost every other subject, there is evidently some cause for surprise that so little has been attempted in this direction. It might be suggested that those who are able to present such works to the public in an attractive form, and yet withal correct in their details, and useful in their ultimate results, consider it a little below the dignity of their vocation, or beyond the legitimate scope of their profession; or it may be, that there still remains in them a small amount of that old conservative spirit (which, by the by, can scarce be the true spirit of conservatism), which fears the extension of knowledge, or dreads the consequence of science made popular. This is, at the least, but to create a monster after the fashion of Frankenstein, and to be alarmed at its shadow. Whatever may be the cause, the fact still remains; and whilst the study of chemistry is becoming daily more diffused, anatomy and physiology, the laws of health and disease, are forgotten, at the same time as the line of the poet—

"'The proper study of mankind is man.'

"There cannot be a doubt that medical men are the persons best qualified to make the public acquainted with these subjects; but they appear to look at the work with fear and trembling, lest in its performance they should be deemed empirics and charlatans. A little reflection would serve to convince them that their fears are groundless, and that they leave a *duty* unperformed in not taking the task out of the hands of those less fitted to render it the justice it deserves. There are many vulgar errors to be exploded, many wrong notions to be set right, and there is much room left for sound instruction, which in the end will not diminish the need of medical men, nor decrease the limits of their practice. On the contrary, a correct appreciation of the dangers of delay, and a ready recognition of the early stages of disease, will send sufferers sooner in search of good medical advice, than while under the spell of ignorance, when slight indispositions are ascribed to all imaginable causes but the right, and the most absurd steps are taken to abate maladies which have no existence save in imagination and the fabulous history of the past. Not that we intend to advocate the publication of a series of handbooks on domestic medicine, or to recommend how one reader may physic his friend, or operate upon his neighbour; but rather that he may understand, appreciate, and respect the house in which his spirit dwells, and know when it is getting out of order; the consequences liable to ensue; and how he may preserve himself from such forms of disease as may result from either ignorance or carelessness. How often do we meet with cases in which we are compelled to give expression to our regret that the patient had not earlier become sensible of the encroachments of disease? How often have we to say, Why did you so long defer an application for medical advice, and allow your disease to gain so firm a hold upon you? Such remarks are common enough, and much more common than they would be if a better knowledge of the laws of health were more widely diffused. We rejoice that general attention is being directed to these subjects as being in every sense worthy of forming a portion of the elementary instruction to be imparted to our children in the public schools; and when steps are in progress to organize systematic instruction on these points for children, it seems an anomaly that those who have grown up without obtaining such a knowledge, should be forgotten, and no steps taken to remedy the deficiency.

"It may be argued, and with much consistency, that this is the only true means whereby the army of charlatans in medicine will be defeated. Diseases are not as formerly so much imputed to the influences of the heavenly bodies. Nor is their cure supposed to depend upon the movements of the planets; yet errors equally superstitious, if not equally gross, are daily committed. We might refer to some of the malpractices in connexion with diseases of the ear to which reference has been made in the foregoing pages, in confirmation of this statement; and indeed there is scarce a form of disease which has not its popular form of treatment, based more or less on some traditional success, honoured perhaps in its antiquity, but which would be much more 'honoured in the breach than the observance.' The belief in panaceas and 'pathies' of all kinds will vanish before the spread of an enlightened knowledge of physiology and the unchangeable laws which govern health and disease."

*Transactions of the Epidemiological Society of London.* Vol. i. pt. 1. London: Richards. 1860. pp. 128.—We are most glad to receive the Transactions of the Epidemiological Society as a separate work. We think the Society has done well in thus giving to its proceedings a more definite literary form, and we augur highly for the value of its future Transactions from the specimen now before us. This part, in addition to the President's Address at the opening of the Session 1859-60, contains also the following papers:—On the Theory of Zymosis, by Dr. A. W. Richardson; Report of the Diphtheria Committee; Suggestions for Utilizing the Statistics of Disease among the Poor, by Dr. Milroy; and Remarks on the Topography and Diseases of the Gold Coast, West Coast of Africa, by Robert Clarke, Esq., late of H. M. Colonial Medical Service. Each of these papers presents points of unusual interest, and, in addition to them, we would especially note the valuable report on the progress of epidemics in 1859, by Dr. MacWilliam, C.B., F.R.S., the Secretary to the Society, incorporated in the Address of the President. We regret that, in consequence of the late period to which our notice of the Transactions has been deferred, we cannot do more than briefly direct attention, not only to this report, but also to the different papers.

*On Some of the Medico-Legal Relations of the Habit of Intemperance.* By Robert Christison, M.D., F.R.C.P.E., V.P.R.S.E., Senior Ordinary Physician to Her Majesty in Scotland, Professor of Materia Medica in the University of Edinburgh, &c. Edinburgh: A. and C. Black. 1861. pp. 60.—In this pamphlet Dr. Christison has made public a Lecture which he delivered at the Conversazione of the Royal College of Surgeons of Edinburgh on the 19th of March. 1858. The subject is one the importance of which could not well be exaggerated, and one that has been pretty frequently dealt with in the pages of the previous series of this Journal. Dr. Christison's opinions on the questions raised cannot fail to exercise great influence over both professional and public feeling in the matter, and we shall give them space commensurate with their weight rather than with the modest aspect of the pamphlet through which they are made known.

"There is," writes Dr. Christison, "in the present condition of society, a great defect in the treatment of the habitually intemperate, whether we look to the care of their health and person, or to the charge of their affairs and property. And in the profession of the law, while I have recently met with individuals well qualified to judge of the matter from its legal side, who are disposed to arrive at the same conclusion, I have on the other hand ceased of late to encounter anywhere that summary dismissal of the subject with which it used to be disposed of by my legal friends no longer than fifteen or twenty years ago."

The prejudice which lawyers entertain to any extension of legal power for the control of drunkards, Dr. Christison thinks is dependent:—

"*First*, on the wholesome unwillingness of the legal mind to discover defect in the statutes or their administration; *secondly*, on the rarity with which the members of the law are brought into direct contact with cases of habitual intemperance in its bearings upon law; *thirdly*, on the intricacy with which habitual intemperance, as a disease, is interwoven with habitual intemperance as a vice, and the natural repugnance of law no less than common sense, that the vice of intemperance should be dealt with in any other way than by moral suasion and religious influences; and *fourthly*, on the tendency of lawyers in common with the educated public at large, to regard insanity too much as a mere object of coercion, and not like medical men, as rather a subject of treatment for which coercion is nothing else than a necessary condition."

Conceiving that there is "no fitter way of explaining the relations of intemperance to insanity, and through insanity to the civil law, than by presenting the views entertained on those subjects by the medical profession," Dr. Christison proceeds to describe the "leading phenomena of habitual intemperance from the simplest form of intoxication without any other manifest disturbance of the mind, to that in which the fit ends with simple delirium tremens; and then to the complication, in which delirium tremens in its turn gives place to mere weakness of mind, or to maniacal delusions or to ordinary general mania."

He next forcibly sketches the blending of the phenomena of habitual drunkenness with those of ordinary mania and the embarrassment arising therefrom in a legal point of view. He then proceeds:—

"On considering attentively the several varieties of disturbance of the mind which may thus occur in connexion with the habit of frequent excess in alcoholic liquors, it will evidently appear that there are many varying modes and degrees in which these mental states must be apt to affect the civil rights and other legal relations of the persons who suffer from them. Regarding some of these influences I am not quite competent to form or pronounce an opinion. But I believe they will be all easily settled in the hands of any lawyer of ability, if law and medicine could come to agreement on a question in which medical men, as exercising the healing art, are first concerned, but lawyers not less so, as it deals with the most important of all civil rights—personal liberty, and the privilege of doing what one likes with his own.

"Without some modification of the law, or at least a change in the mode of applying it, in respect to the personal liberty and the managment of the affairs of habitual drunkards, it is impossible for medical men to subject such persons in due time to the proper treatment for their cure, or to ensure its due continuance. This is one great defect for which medical men earnestly crave a remedy. But at the same time it is quite evident that, if this question be satisfactorily settled, other points also will be equally settled—such as the public safety, the safe custody of the individuals themselves, the security of their affairs, the happiness and well-being of their families. Nor will the lawyer, in such circumstances, experience any great difficulty in laying down principles for determining how far persons so situated must submit to curtailment of their right to enter into various civil contracts.

"In that form of the effects of intemperance in which the craving for strong drink passes through the stage of delirium tremens into ordinary general mania, there can, of course, be no doubt of the necessity of some such complete restraint as an asylum and a curator. There can be scarcely more doubt about the last form which I have described, in which sundry delusions become permanently fixed, some of them dangerous. Even when mere feebleness of intellect during the intermissions between the fits of drinking is very great, treatment is not easy except in an asylum; and certainly the subject of such infirmity cannot take due care of himself, his family, or his affairs.

"On the other hand, in the moderate forms of simple repeated brief intoxication, which pass by insensible shades into the case of mere slight irregularities, opposed to modern manners, but not referrible to an intense craving, interference of the law in any shape is to be deprecated, and would be denied. And yet we must not lose sight of the undoubted fact, that such minor shades of avidity for intoxicating liquors, which, amidst the improved habits of the present day, will make any man painfully conspicuous, are too often the sure prelude of what is to become, ere long, an insatiate ungovernable craving.

"But, thirdly, there is a great body of habitual drunkards who are placed in the mean between these two extremes; and for whom perfect liberty and rigorous confinement are alike unsuitable. To this denomination belong the continuous and periodical drunkards, who sustain no other perturbation of their faculties than frequent and protracted intoxication,—many of those who have also, in addition, delirium tremens, but nothing further,—and some who, in the intermissions, are likewise affected with only a moderate degree of feebleness of mind, or with some harmless monomaniacal delusion. Many such drunkards have so great an amount of intelligence during the intermissions between their drinking-bouts, that they can mingle with decency in society, and even apply themselves successfully to their ordinary vocations. To them, confinement in an asylum, if in some respects useful, is hurtful in others. For the mind very soon recovers its healthy tone under the discipline of an asylum; repugnance to confinement invariably follows; and the straining against restriction sometimes positively impedes the progress of cure of the fundamental disorder. Besides, such persons really thus cease soon after admission to come under the provisions of the statute which authorizes detention in an asylum on account of lunacy.

"It is for this numerous class that physicians anxiously desire that another kind of restraint be legalized, for the sake of their cure. It is important, however, to bear in mind, that the legislation of a modified restraint for that end would also fix a defined landmark for regulating the law in the application of other checks of a purely legal bearing."

After treating briefly of Salvatori's topographical description of habitual drunkenness, Dr. Christison adds—

"Having advanced thus far, I may be asked to define what constitutes drink-madness. This, however, I will decline. I fear that, in an attempt to do so, we should lose sight of facts in the mazes of logic, and the clouds of psychology. But, as a practical rule for both lawyer and physician, I will venture to state the proposition that, when in a particular case the avidity for strong liquors has reached such a height as—1. To cease to be controllable by every plain and powerful moral and religious consideration; 2. To overwhelm the mind in frequent or continual intoxication; and 3. To occasion danger or actual damage to one's affairs, or family, or both—it ought to be regarded as a disease, and treated as an insanity.

"It has been urged, as an objection to treating the ungovernable avidity for strong drink as a disease and an insanity, that a distinction cannot be drawn between intemperance the disease, and the vice intemperance. I do not, however, exactly comprehend where lies the objection. The distinction is impossible in theory, because we have seen that the disease may originate in the vice, and the transition from the latter to the former is almost imperceptible. But whence the necessity for a distinction at all? If it be right to cure the disease, it is surely not less so to cure the vice. And it ought to be satisfactory, rather than otherwise, that both ends will be simultaneously attained by the same means.

"Equally unpractical and futile is the objection that we cannot draw the boundary between the degrees of the disease which require restriction of liberty, and those which must be admitted to be too slight to warrant any interference of the kind. For, in the first place, there is no other form of insanity which is not similarly circumstanced: secondly, however difficult it may be to draw the boundary in an abstract definition, the difficulty cannot be great in actual practice, when it is an individual case we have to deal with: and thirdly, if in doubtful circumstances liberty should chance to be invaded on somewhat too slender grounds, this is one of the rare instances in law proceedings in which it is not easy to say what harm can arise. Who suffers? The public, or the family, or other relatives, or the party's affairs, or the party himself, or the dignity of the law? Not one of them. They may, and will, and often do suffer from too lax, but never from too strict an application of the practice which it is the object of the preceding observations to recommend."

Dr. Christison, in conclusion, offers the following suggestions applicable to Scotland:—

"*In the first place,* It is by no means necessary, in any legislation which may be thought advisable, either to define this disease, or even to mention it. To the already settled opinion of the medical profession, and to the growing conviction among the sheriffs of counties, and other law officers who are brought directly in contact with it, may be safely left the farther recognition of it as an insanity requiring treatment as such.

"*Secondly,* It ought to be made lawful for the nearest relatives to send the inveterate drunkard—somewhat according to the form in cases of ordinary lunacy, viz., under certification of two medical men and warrant of the sheriff—not to an asylum—but to such an establishment as that on the island of Skye. I do not mean that all such establishments must command equal advantages with that of Skye; but the nearer to it in that respect, so much the better. Especially, however, is it essential that there should be ample space for free and safe exercise and amusement; and, therefore, that no dealer in strong drink should be within easy reach; and where an establishment has been set down in a place possessing that exemption, it ought to be not lawful for a bench of justices, with perhaps a spirit-dealer or two among their number, to license, as I have known done in circumstances somewhat analogous, a vendor of wines and British spirits within convenient distance.

"*Thirdly,* All such sanitaria should be licensed by the sheriff of the county, on proof that the appropriate conditions will be secured, and under his approbation of the main rules of management; and they should be visited from time to time by the sheriffs and lunacy inspectors.

"*Fourthly,* When any one is sent to such an establishment, he ought not to be dismissed for less than six months, except for special reasons, satisfactory to the sheriff,—it being well ascertained that no man was ever effectually broken of the habit of excess in a shorter period. After that, let him be dismissed, if the relatives and the proprietor of the establishment be satisfied of the probability of good conduct. If they are not satisfied, the sheriff's consent should be necessary.

"*Fifthly,* The proprietor of such an establishment should have the right of ordering any inmate who seriously infringes the fundamental rules to be confined to his room—or the immediate precincts of the house—for a week; such order to be intimated to the sheriff, who may visit, or order a visit, should he see cause. And the inmate should be removable into an asylum on easy conditions, when circumstances render treatment in the sanitarium inadequate, and the more severe restraints of an asylum indispensable.

"*Sixthly,* When an ungovernable drunkard is sent to a sanitarium, it is by no means necessary in all cases that he should be entirely deprived of the management of his affairs. But it would be of moment that he should conduct them only with advice of one nominated for the purpose by the sheriff, as soon as possible after his admission. Should that arrangement prove inadequate, it will be left to the relatives to sue for the appointment of a curator on the ordinary footing.

"As the present propositions only contemplate the treatment of those whose own means, or whose relatives, will defray the cost, it is unnecessary to provide for the erection of sanitaria. They will arise fast enough through private enterprise under the required modification of the recent Lunacy Act."

*Nephalism, the True Temperance of Scripture, Science, and Experience.* By JAMES MILLER, F.R.S.C., Surgeon in Ordinary for Scotland to the Queen, and to H.R.H. the Prince Consort. Glasgow: Scottish Temperance League. 1861. pp. 213.

This is another of Mr. Miller's brilliant works in the service of Temperance. The substance was originally delivered in the form of lectures to the students of the University of Edinburgh. The title is derived from "Νηφω, I do not drink wine or strong drink; νηφαλιος, without wine or strong drink; νηφαλισμος, the condition of being without wine or strong drink, or true sobriety." We heartily commend the work to all persons young or old.

*The Present State of the Medical Profession in Great Britain and Ireland, with Remarks on the Preliminary and Moral Education of Medical and Surgical Students: a Book that will be found Helpful to Medical Students, and the Parents and Guardians of Young Men, and of General Interest to the Members of the Medical Profession.* By WILLIAM DALE, M.R.C.S., L.S.A., Undergraduate of the London University, &c. London: A. W. Bennet. 1860. pp. 70.

A sober, thoughtful pamphlet, the object of which is best expressed by its title-page.

The following reprints lie on our table:—

*The Scientific Place and Principles of Medical Psychology, an Introductory Address,* by Professor Laycock; *On the Proximate Analysis of Plants and Vegetable Substances,* by Dr. Frederick Rochleder, translated from the German by W. Bastick; *Medical Psychology,* by Robert Dunn, F.R.C.S. Eng.; *Clinical and Pathological Notes on Pericarditis,* by Dr. W. F. Gairdner; *The Legal Relations of Insanity,* by Dr. David Skae; *Cottage Asylums* and *Endemic Degeneration,* by Dr. W. A. F. Browne; *The Modern Pathology and Treatment of Venereal Diseases,* by Dr. P. H. Watson; *On "Supporting the Perinæum,"* by Dr. Graily Hewitt; *The Cottage System and Gheel,* by D. Sibbald; *An Inquiry into a frequent Cause of Insanity in Young Men,* by Dr. Ritchie. Also the second edition of a Lecture on *Ale, Wine, Spirits, and Tobacco,* delivered before the Leicester Literary and Philosophical Society by Dr. Barclay; and a Paper on *Diphtheria,* read before the New York Academy of Medicine by Dr. James Wynne.

